# Microsatellites in the Genome of the Edible Mushroom, *Volvariella volvacea*

**DOI:** 10.1155/2014/281912

**Published:** 2014-01-19

**Authors:** Ying Wang, Mingjie Chen, Hong Wang, Jing-Fang Wang, Dapeng Bao

**Affiliations:** ^1^National Engineering Research Center of Edible Fungi and Key Laboratory of Applied Mycological Resources and Utilization, Ministry of Agriculture and Shanghai Key Laboratory of Agricultural Genetics and Breeding and Institute of Edible Fungi, Shanghai Academy of Agriculture Science, Shanghai 201403, China; ^2^Key Laboratory of Systems Biomedicine, Shanghai Center for Systems Biomedicine, Shanghai Jiao Tong University, Shanghai 200240, China

## Abstract

Using bioinformatics software and database, we have characterized the microsatellite pattern in the *V. volvacea* genome and compared it with microsatellite patterns found in the genomes of four other edible fungi: *Coprinopsis cinerea*, *Schizophyllum commune*, *Agaricus bisporus,* and *Pleurotus ostreatus*. A total of 1346 microsatellites have been identified, with mono-nucleotides being the most frequent motif. The relative abundance of microsatellites was lower in coding regions with 21 No./Mb. However, the microsatellites in the *V. volvacea* gene models showed a greater tendency to be located in the CDS regions. There was also a higher preponderance of trinucleotide repeats, especially in the kinase genes, which implied a possible role in phenotypic variation. Among the five fungal genomes, microsatellite abundance appeared to be unrelated to genome size. Furthermore, the short motifs (mono- to tri-nucleotides) outnumbered other categories although these differed in proportion. Data analysis indicated a possible relationship between the most frequent microsatellite types and the genetic distance between the five fungal genomes.

## 1. Introduction

*Volvariella volvacea*, the Chinese straw mushroom, is an edible, straw-degrading, basidiomycetous fungus that has been cultivated for over 300 years. Currently ranked third in terms of production worldwide [1, 2], this mushroom has commercial importance that continues to increase due to its delicious flavor and texture, nutritional attributes, medicinal properties, and short cultivation cycle. *V. volvacea* is rich in protein, essential amino acids, vitamin C, and other bioactive components [3, 4]. According to traditional Chinese medicine, consuming the mushroom is good for the liver [5] and stomach, relieves summer heats, and enriches milk production in women following childbirth. Furthermore, antioxidants from *V. volvacea *are reported to enhance immunity, reduce cholesterol levels, and prevent atherosclerosis [6]. *V. volvacea *also plays an important ecological role by degrading the various agricultural wastes such as rice and wheat straw, cottonseed hulls, sugar cane bagasse, oil palm pericarp, banana leaves, and other carbonaceous materials used for cultivation [3, 7]. However, in spite of these nutritional, medicinal, and environmental benefits, relatively little is known about the molecular biology of this mushroom.

Microsatellites, also known as simple sequence repeats (SSRs) or short tandem repeats (STRs), are 1–6 base-pair nucleotide motifs, repeated in tandem at least five times. They are distributed in both coding and noncoding regions of eukaryotic and prokaryotic genomes [8, 9] and exhibit high levels of polymorphism. Since the late 1980s, many microsatellites have been used as genetic makers for species identification [10] and classification and in genome fingerprinting and mapping studies [11–14]. Subsequent research has revealed that microsatellites are involved in gene regulation, and organism development and evolution [15, 16]. In most cases, the effects of microsatellites are determined by their genomic location. For example, mutations to microsatellites located in coding and promoter regions lead to phenotype modification [17–20], while in 5′-untranslated regions (5′-UTRs) they affect gene transcription or regulation. In intron regions, microsatellite mutations impact gene transcription, regulation, mRNA splicing, and gene silencing, and in 3′-UTRs they are involved in gene silencing and transcription slippage [16, 21]. Moreover, since the frequency of these elements in genomes is proportional to the genome coverage, they can help explain how genomes are organized [11]. However, there are major drawbacks associated with microsatellite research including the time and costs associated with isolating microsatellites from the whole genome [22], a process that involves the screening of small insert genomic DNA libraries or the construction of SSR-enriched libraries [23]. However, our previous research into the degradation and sexual reproduction mechanisms adopted by *V. volvacea* has resulted in the sequencing of the whole genome [1], thereby facilitating genome-wide analyses of microsatellite distribution.

In this study, the entire genome of a monokaryotic *V. volvacea* strain (V23-1) has been screened to determine the distribution and density of microsatellites in different genomic regions. Particular emphasis has been given to microsatellites located in genes with molecular functions, and microsatellites in the *V. volvacea *genome have also been compared with those present in the genomes of four other edible fungi. For this purpose, we designed 100 primer pairs based on identified microsatellites loci used for genetic mapping. Our data will serve to establish the functional and evolutionary significance of these sequences and contribute to their future use as molecular markers.

## 2. Materials and Methods

The complete genome sequence of *V. volvacea* strain V23 was downloaded from the FTP site of GenBank. Information relating to the location of the gene models, introns, coding sequences (CDSs), 5′-untranslated-(5′-UTRs), 3′-untranslated-(3′-UTRs), and intergenic regions were obtained from the *V. volvacea *genome study group. 5′-UTRs are defined as the sequence located between a transcription start point and the beginning of the start codon of the transcript. 3′-UTRs are defined as the sequence between the stop codon and the last base of the transcript. Except introns, CDSs and UTRs, all the other regions in the genome are classified as intergenic regions. *Coprinus cinereus*, *Schizophyllum commune*,* Agaricus bisporus*, and* Pleurotus ostreatus* genome sequences used in this study (Table S1) (See Supplementary material available online at http://dx.doi.org/10.1155/2014/281912) were downloaded from the Joint Genome Institute (JGI) website.

MISA software (http://pgrc.ipk-gatersleben.de/misa/) was used to locate and identify both perfect microsatellites and compound microsatellites interrupted by a certain number of bases. Mono- to hexanucleotide microsatellite motifs were identified using the following default parameters: mono- with at least 10 repeats; di- with at least six repeats; tri-, penta-, and hexa- with at least five repeats; the maximum number of bases between two microsatellites was 100 bp. [18, 24]. Unit patterns of repeats with circular permutations were considered as one type for statistical analysis. The same conditions were used to identify microsatellites in all the genome assemblies [25, 26]. To more accurately compare all the repeat types existing in different genomic regions, the relative abundance (mean of the number of microsatellites per Mb of the sequence analyzed) and the relative density (mean of the microsatellite length in bp per Mb of the sequence analyzed) were calculated separately [27]. Since longer microsatellites may display higher levels of polymorphism, primers for these loci were designed using the Primer3 software (http://frodo.wi.mit.edu/primer3/).

Gene models having microsatellites in exons, introns, and UTRs were isolated and then scanned for InterPro domains and gene ontology (GO) annotation. The latter was used to assign each gene-encoded protein to one of the three defined categories (molecular function, biological process, or cellular component), and WEGO (Web Gene Ontology Annotation Plot) [28] was used to plot the GO annotation data.

## 3. Results

### 3.1. Identification and Location of Microsatellites in the *V. volvacea *Genome

The *V. volvacea *genome contained a total of 1346 microsatellites, with a relative abundance of 38 microsatellites per Mbp (Table 1). Microsatellites with periods ranging from 1 to 6 (i.e., mono- to hexa-) accounted for 57.4% (773), 8.2% (110), 29.7% (400), 1.4% (19), 1.0% (14), and 2.2% (30) of the total, respectively. From these, 100 microsatellite loci were selected and 100 primers were designed accordingly (Table S2).

The entire genome (35.72 Mb) was divided into five regional types consisting of 5′UTRs (0.69 Mb), CDSs (17.42 Mb), introns (5.71 Mb), 3′UTRs (1.6 Mb), and intergenic (10.31 Mb). Microsatellites were more numerous in the intergenic regions with a relative abundance of 58 No./Mb (Table 1). Over 50% of the mono-, di-, and tetranucleotides were located in the intergenic regions, whereas tri- and hexanucleotides appeared more frequently in the CDSs. Pentanucleotide microsatellites were fairly evenly distributed within the CDSs, introns, 3′UTRs, and intergenic regions. No penta- and hexanucleotides were found in the 5′UTRs, and no tetranucleotides were found in CDSs.

**Figure 1:**
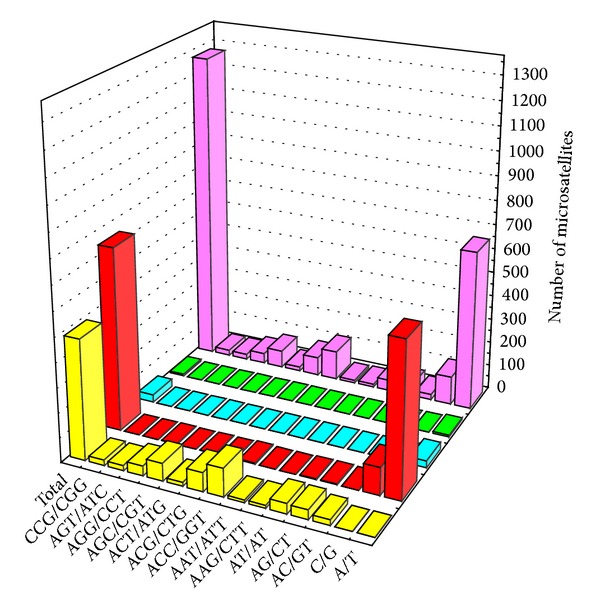


The fourteen most abundant microsatellite types (≥10 motifs) detected in the *V. volvacea *genome constituted 94.7% of the total (Figure 1). A/T occurred at the highest frequency (51.6%), followed by ACC/GGT (9.34%), and C/G (9.11%). With the exception of the mononucleotide motifs, the majority of these most abundant microsatellites motifs were repeated less than 10 times. Only 13 (1%) microsatellite motifs exhibited a large repeat number. Trinucleotides were the primary types among the above fourteen most abundant microsatellites types, but their distribution was highly variable with a wide range (from 0.86% to 9.34%) of frequencies. In addition, there were fifteen tetranucleotide types, twelve pentanucleotide types and twenty-five hexanucleotide types identified within the whole genome, but none of these contained more than ten different motifs. Among the fourteen most abundant microsatellites types (Figure 1), the longest motifs were T (61 bp), CAC (42 bp), GAG (39 bp), TGA (30 bp), AAT (27 bp), AAG (24 bp), ACG (24 bp), TCA (24 bp), CTG (24 bp), AT (22 bp), C (21 bp), AG (20 bp), CCG (18 bp), and GT (14 bp), respectively.

### 3.2. Distribution of Microsatellites in the *V. volvacea* Gene Models and Functional Properties of the Genes Containing Microsatellites

Of the 11084 genes identified in the whole genome of *V. volvacea*, 649 (5.9%) contained 748 microsatellites, with 72 of these containing more than one microsatellite. The relative abundance of microsatellites in the gene models was 29 No./Mb. Altogether, 365 microsatellites were detected in the CDSs of 323 genes (2.9% of the total), contributing 27.1% of the total in the entire *V. volvacea *genome. Trinucleotides (299) were the most abundant category (81.9%) in all the gene models and, with the exception of the mononucleotide microsatellites, the trinucleotide CAC was the most frequent (with 35 motifs), followed by CAG (24 motifs), GAC (20 motifs), and CCA (16 motifs). All these motifs encoded aliphatic amino acids such as valine, leucine, and glycine. InterPro and KEGG database scanning revealed that, of the 649 gene models containing microsatellites, 365 contained at least one known domain, 190 participated in a biological pathway, and 147 had been annotated definitively. These genes encoded proteins including cytochrome P450 monooxygenases, carbohydrate-degrading enzymes, kinases, dehydrogenases, and transport proteins. Thorough analysis of the microsatellite distribution and motif type among the 147 annotated genes revealed that the microsatellites were more frequent within the CDS regions of the kinase genes and that trinucleotides were the most abundant motif (Table S3). However, these conditions did not apply to other gene categories. In terms of molecular function, the annotated genes included three with electron carrier activity, 23 with catalytic activity, five involved in binding, two with structural molecule activity, and two with transporter activity (Table 2). Some genes contained more than one microsatellite. For example, four different types of microsatellite motifs, (GAC)5, (GCT)5, (GAC)5, and (CCGCAC)6, were detected in the CDS of the CAMK/CAMK1 protein kinase gene. The Ca^2+^ transporting ATPase gene also contained four microsatellites, three in the CDS and one in the intron regions, while two or three microsatellites were identified in either the CDS or intron regions of other genes.

**Figure 2:**
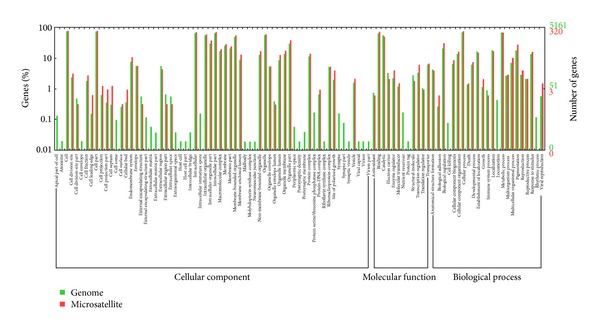


The whole genome of *V. volvacea *and the genes having microsatellites were categorized on the basis of their homologous gene function by the Gene Ontology Consortium. Gene ontology numbers for the best homologous hits were used to determine molecular function, cellular component, and biological process ontology for these sequences. An inferred putative gene ontology annotation was found for 5161 genes in the genome, of which only 320 (254 associated with cellular components, 243 with biological processes, and 242 with molecular function) contained microsatellite loci. The GO terms in the three ontologies of the whole genome totaled 105, with 73 in the genes having microsatellites. In the cellular component ontology, the cell (76.2% in whole genome and 79.4% in genes containing microsatellites) and the cell part (76.2% and 79.4%) were the two main types of GO term, followed by the organelle (56.8% and 60.9%) and organelle part (30.8% and 38.8%). In the biological process ontology, cellular process (69.9%, 75.9%) and metabolic process (70.1%, 68.8%) were the two main GO terms, and in the Molecular Function ontology, the functions of the major components were binding (65.5%, 75.6%) and catalytic activity (58.8%, 52.5%). Compared with the whole genome, some GO terms were not present in genes having microsatellites, including cell apex (GO:0045177), external encapsulating structure (GO:0044462), intracellular immature spore (GO:0042763), protein serine/threonine phosphatase complex (GO:0008287), synapse (0045202), nutrient reservoir activity (GO:0045735) and locomotion (GO:0040011), (Figure 2). Most of the absent GO terms were concentrated in the cellular component ontology.

### 3.3. Comparison of Microsatellite Distribution in the Genomes of *V. Volvacea *and Four Other Edible Fungi

The number and types of microsatellites in the *V. volvacea *genome and four other fully sequenced edible fungal genomes that varied in size from 30.2 Mb (*Agaricus bisporus*) to 38.6 Mb (*Schizophyllum commune*) were compared (Table 3). Microsatellite content in these species was not directly proportional to the size of the genome since *A. bisporus* contained the highest number (3134, relative abundance 103.8 per Mbp) compared with only 1206 in *S. commune* (relative abundance 31.2 per Mbp). The *C. cinereus *and *P. ostreatus* genomes contained 2050 and 1314 microsatellites, respectively. However, although *V. volvacea* and *P. ostreatus* showed the same relative abundance of microsatellites (38 per Mbp), the relative densities were different because the total length of microsatellites in *P. ostreatus *was longer. Yet, based on the comparison of microsatellites in the wholegenomes, the microsatellite content in the *V. volvacea *genome exhibited closer similarity to *P. ostreatus *and *S. commune *than to the *A. bisporus *and* C. cinereus*.

**Figure 3:**
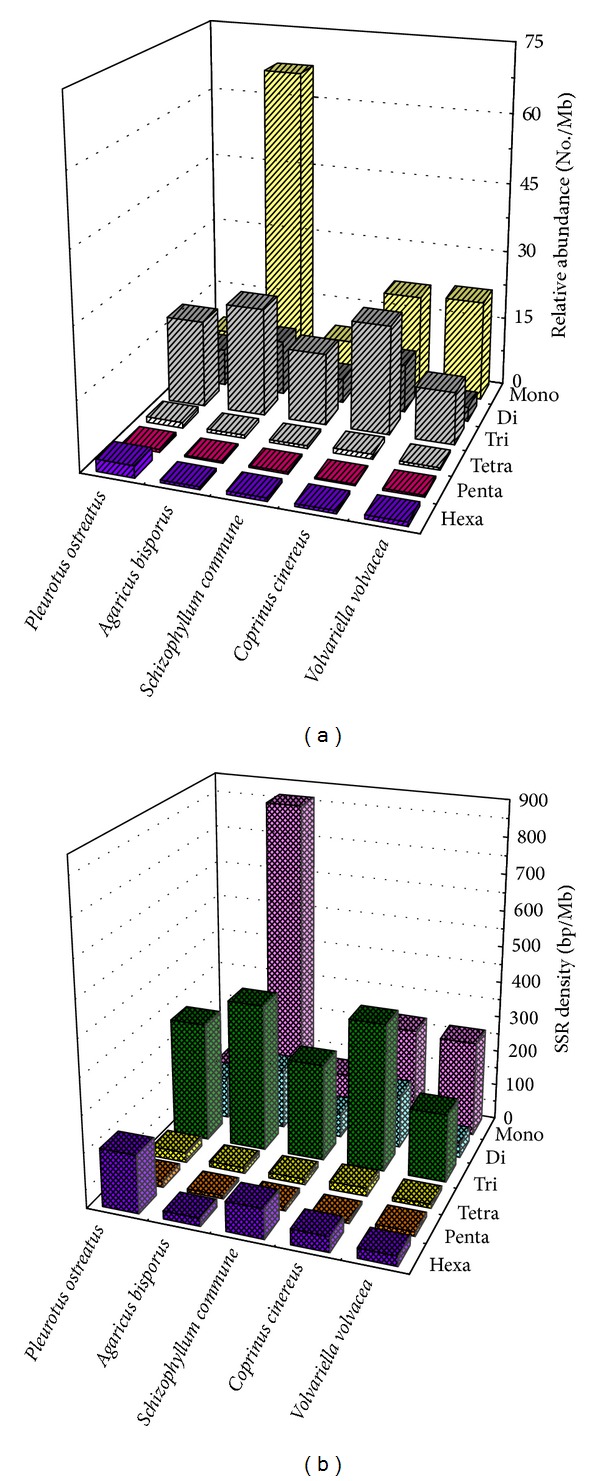


**Figure 4:**
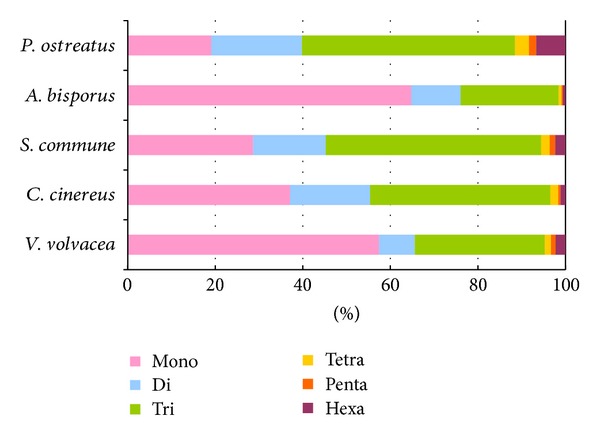


The five fungal genomes exhibited considerable differences with respect to the number, relative abundance, and relative density of mono-, di-, tri-, tetra-, penta-, and hexanucleotides (Table 4, Figure 3). For example, mononucleotide motifs outnumbered all other microsatellite classes in the *V. volvacea* and *A. bisporus* genomes, with 2030 (64.8% of the total) detected in the latter. However, trinucleotide microsatellites (followed by mononucleotides) were the most common motifs in *S. commune* and *C. cinereus*, while trinucleotide microsatellites (followed by dinucleotides) were most frequent in *P. ostreatus*. Comparison of the relative abundance (Figure 3(a)) and the relative density (Figure 3(b)) of the six microsatellite categories revealed general agreement except in the case of the *P. ostreatus* genome where hexanucleotide microsatellites were infrequent but relatively dense. In contrast to the clear disparity in the total number of microsatellites in the *V. volvacea* and *A. bisporus* genomes, the proportion of the six microsatellite categories was very similar (Figure 4).

**Figure 5:**
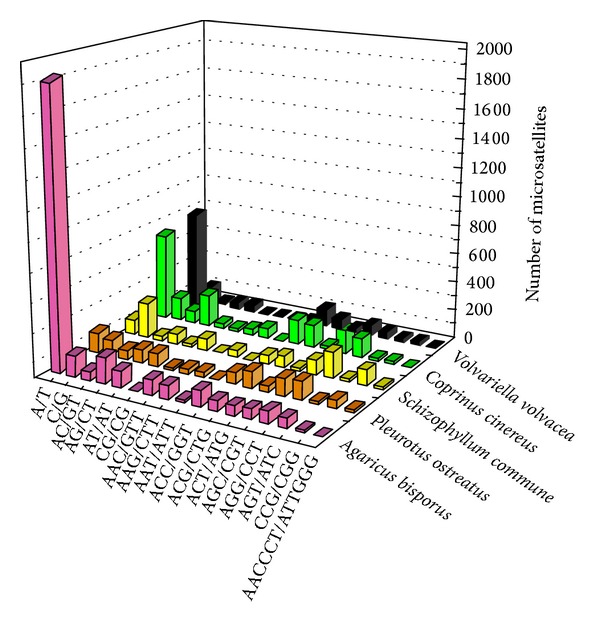


The number of motif types of 17 different microsatellites in each of the five mushroom species is shown in Figure 5. The mononucleotide A/T was the most frequent in the majority of species, and only the *S. commune* genome contained more C/G than A/T. Of the dinucleotide motifs, CG was common in *C. cinereus*, *S. commune, *and *P. ostreatus* but least frequent in *V. volvacea *and *A. bisporus*. Conversely, the trinucleotide AAT/ATT was comparatively common in *V. volvacea *and *A. bisporus* but rare in *C. cinereus*, *S. commune, *and *P. ostreatus*. Furthermore, only eight AAC/GTT trinucleotide motifs were detected in *V. volvacea *and in *S. commune* compared with more than 35 in *C. cinereus*, *P. ostreatus*, and *A. bisporus*. Tetra-, penta-, and hexanucleotide SSR densities were very low and only the hexanucleotide AACCCT/ATTGGG was relatively common with at least 10 motifs identified in both *S. commune* and *P. ostreatus*. The longest motifs varied from 34 repeats of the dinucleotide AG/CT and the hexanucleotide AACCCT/ATTGGG in *A. bisporus* and *C. cinereus*, respectively, to 61 repeats of the mononucleotide A/T in *V. volvacea *(Table 5). AACCCT/ATTGGG was also the longest microsatellite identified in *S. commune* and *P. ostreatus* with 36 and 38 repeats, respectively.

## 4. Discussion

Microsatellites have contributed significantly to studies in population genetics [29, 30] and molecular ecology [11], have served to explain the phenomenon of genome expansion in certain species [31], influenced the expression of quantitative genetic traits [32], and have been used to analyze the human genome for human diseases [33, 34]. Considerable progress has been made in microsatellite development, including associated bioinformatics. For example, several studies have focused on the basic distribution patterns and diversity across whole genomes to better understand the role of microsatellites. However, since relatively little comprehensive analysis of microsatellites in the genomes of edible fungi has been undertaken, we have used computational analysis to characterize and compare the microsatellites in the entire genome of *V. volvacea* and the genomes of four other edible fungi (Table S1). Information on the relative abundance of these microsatellites, combined with the distribution patterns in both the coding and noncoding regions of the genome, may provide clues to the functionality of microsatellites in gene regulation.

At present, there is no standard cut-off limit for the minimum length of microsatellites [35]. Adopting slippage rate changes of around 10 bases for mono- and dinucleotide repetitions, universal thresholds of 8–10 bp and 7–10 bp were proposed for mononucleotide microsatellites in yeast [36] and eukaryotes [37], respectively. However, the threshold for human microsatellites was found to depend on their motif size (9 repeats for mononucleotide and 4 repeats for di-, tri-, and tetranucleotide microsatellites) [38]. Accordingly, and in order to compare our data with previous studies of other fungi, we identified mono-, di-, tri-, tetra-, penta- and hexanucleotide microsatellite motifs at minimum repeat numbers of 10, 6, 5, 5, 5, and 5, respectively. Of the 1346 microsatellites identified, 72.9% were embedded in noncoding DNA (corresponding to 51.23% of the genome assembly), and 61% were located in the intergenic regions (28.9% of the genome assembly). This distribution pattern, which is common in fungal genomes, has been attributed to negative selection against frame-shift mutations in coding regions [39] and possibly accounts for the small number of microsatellites in fungi. Another contributing factor is the relatively smaller amount of noncoding DNA in fungi, due to the high density of genes within the fungi genome, compared with higher eukaryotes. This distribution implies that microsatellites are generated in intergenic regions by duplication or transposition.

In addition to possible relevance as evolutionary neutral DNA markers [40], microsatellites have some functional significance, including effects on chromatin organization, regulation of gene activity, recombination, DNA replication, cell cycle, and mismatch repair systems [41, 42]. Firstly, we can estimate the function of a microsatellite by its position. Judging from previous experience, microsatellites located in CDSs can alter the function of the protein [20], those located in introns can affect gene transcription, those located in 5′UTRs can regulate gene expression, and those located in 3′UTRs may cause transcription slippage [43]. In *V. volvacea*, 55.6% of the total number of microsatellites were scattered in gene models and nearly half of these microsatellites were located in coding regions. Due to the high mutation rate of microsatellites, genes containing microsatellites in their coding regions would not be conserved. Close inspection of the great majority of microsatellites appearing in kinase-encoding genes was located in CDSs, suggesting these genes are capable of undergoing mutation. Microsatellites were also found in the introns, 5′UTRs, and 3′UTRs of CYPs, carbohydrate-degrading enzymes, dehydrogenases, and transport proteins. Earlier studies have shown that microsatellites located in promoter regions may affect gene activity [44]. In addition, some long microsatellites located in intergenic regions may have special functions. For instance, long microsatellites involved in sister chromatid cohesion, which indirectly assist kinetochore formation, are highly clustered in the centromere [45]. Excess numbers of microsatellite repeats may play important roles both in genomic stability and also in the evolution of additional genomic features. Consequently, the longest motifs (T)61, (CAC)14, and (GAG)13 in *V. volvacea* merit close attention with respect to possible functionality.

Comparative analyses of the microsatellite distribution in the genomes of *V. volvacea *and four edible fungi revealed that, in all cases, the majority of the microsatellites were mono-, di-, and trinucleotides, accounting for up to approximately 90% of all the microsatellites identified. However, their respective percentages varied in the different genomes.* V. volvacea* and* A. bisporus* showed a great affinity for mononucleotide repeats compared to the other three genomes in which trinucleotide microsatellites were the predominant type. Furthermore, microsatellites in the *S. commune*, *C. cinereus* and *P. ostreatus* genomes were comparatively longer. This was unexpected, especially in the case of *P. ostreatus *(the second smallest genome of the five), since an earlier study had concluded that microsatellites in larger genomes were longer compared with those in smaller genomes [27]. Hence, neither the abundance nor the length of the microsatellites in these fungi was correlated with genome size. In the case of microsatellite types, few trends were evident. A/T were the most frequent mononucleotide repeats in *A. bisporus*, *V. volvacea*, *C. cinereus*, and *P. ostreatus*, whereas C/G was most frequent in the *S. commune* genome. Among the dinucleotide motifs, AC/GT, AG/CT, and AT/AT were present at higher frequencies in *A. bisporus*, *V. volvacea*, *C. cinereus*, and *P. ostreatus*, whereas CG/CG was the most common in* S. commune*. The reason for the difference may be attributable in part to the higher GC content in the *S. commune* genome and to its distant phylogenetic position.

## 5. Conclusion

Comprehensive analysis of microsatellites in the *V. volvacea* and four other completely sequenced edible fungal genomes will provide better understanding of the nature of these important sequences. Such understanding of the characteristics of microsatellites in the genomes of *V. volvacea* and the other edible fungi will serve many useful purposes including the isolation and development of variable markers and will facilitate research on the role of microsatellites in genome organization.

## Supplementary Material

The detailed information for the fungal genomes involved in the current study, the primers randomly selected from *V. volvacea* genome, and significant microsatellites in *V. volvacea* were available in the Supplementary Materials.

## Figures and Tables

**Table 1:** Number, percentage, and relative abundance of microsatellites in the different regions of the *V. volvacea *genome.

	5′UTR	CDS	Introns	3′UTR	Intergenic regions	Total
Genome size/Mb	0.69	17.42	5.71	1.61	10.31	35.72
Percentage of the genome	1.93	48.77	15.99	4.51	28.86	100
Mono No.	8	32	231	49	453	773
%	1.03	4.14	29.88	6.34	58.60	100
No./Mb	12	2	40	30	44	22
Di No.	1	16	21	2	70	110
%	0.91	14.55	19.09	1.82	63.64	100
No./Mb	1	1	4	1	7	3
Tri No.	6	299	29	15	51	400
%	1.5	74.75	7.25	3.75	12.75	100
No./Mb	9	17	5	9	5	11
Tetra No.	2	—	3	2	12	19
%	10.53	—	15.79	10.53	63.16	100
No./Mb	3	—	—	1	1	1
Penta No.	—	2	4	4	4	14
%	—	14.29	28.57	28.57	28.57	100
No./Mb	—	—	1	2	—	0
Hexa No.	—	16	4	2	8	30
%	—	53.33	13.33	6.67	26.67	100
No./Mb	—	1	1	1	1	1
All SSRs No.	17	365	292	74	598	1346
%	1.26	27.12	21.69	5.50	44.43	100
No./Mb	25	21	51	46	58	38

**Table 2:** Microsatellites in the gene models with some molecular functions in *V. volvacea*.

Molecular function	Gene product	Location
Electron carrier activity	CYP547B1	Intron
	CYP5080B3b	Intron
	CYP627A1	Intron

Catalytic activity	Nonribosomal peptide synthetase 12	Intron
	Protoporphyrinogen oxidase	Intron
	Glycoside hydrolase family 13 protein	CDS
	ATP citrate lyase isoform 2	3′UTR
	Adenylate cyclase	CDS
	Xylanase	CDS
	Pyruvate dehydrogenase	CDS
	Modular protein with glycoside hydrolase family 13 and glycosyltransferase family 5 domains	Intron
	long-chain-fatty-acid-CoA ligase	3′UTR
	Glycoside hydrolase family 18 protein	CDS, intron
	Glycoside hydrolase family 15 protein	Intron
	Glycoside hydrolase family 35 protein	Intron
	AMP dependent CoA ligase	Intron
	Serine palmitoyltransferase 2	CDS
	IMP dehydrogenase	CDS, intron
	Trehalase	Intron
	DNA helicase	CDS
	Exo-beta-1,3-glucanase	Intron
	Glycoside hydrolase family 10 and carbohydrate-binding module family 1 protein	Intron
	Potassium/sodium efflux P-type ATPase	CDS × 3, Intron
	Glycoside hydrolase family 38 protein	Intron
	Sodium transport ATPase	Intron
	Fructose-bisphosphate aldolase	CDS, intron

Binding	Sec7 guanine nucleotide exchange factor	3′UTR
	COP8	CDS
	STE/STE11/cdc15 protein kinase	CDS
	Clathrin-coated vesicle protein	Intron
	Carnitine/acyl carnitine carrier	Intron

Structural molecule activity	Beta-tubulin 2 tubb2	Intron
	Iron sulfur assembly protein 1	Intron

Transporter activity	Urea transporter	3′UTR
	Vacuole protein	Intron

**Table 3:** Overview of the five edible fungal genomes.

	*V. volvacea *	*C. cinereus *	*S. commune *	*A. bisporus *	*P. ostreatus *
Sequence analyzed (Mb)	35.7	36.2	38.6	30.2	34.3
GC contents (%)	48.8	51.67	57.5	46.48	50.94
No. of SSRs	1346	2050	1206	3134	1314
Relative abundance (No./Mb)	38	56	31	104	38
Total length of SSRs (bp)	19347	32601	21538	44690	25265
Relative density (bp/Mb)	541	898	558	1478	737
Genome content (%)	0.05	0.09	0.06	0.15	0.07

**Table 4:** Occurrence, relative abundance, total length, and relative density of microsatellites in the five edible fungal genomes.

	*V. volvacea *	*C. cinereus *	*S. commune *	*A. bisporus *	*P. ostreatus *
Mono					
No.	773	761	346	2030	251
No./Mb	22	21	9	67	7
Length (bp)	9440	9950	4425	26065	3131
Bp/Mb	264	275	115	863	91
Di					
No.	110	375	200	354	272
No./Mb	3	10	5	12	8
Length (bp)	1464	5354	2664	4980	3904
Bp/Mb	41	148	69	165	114
Tri					
No.	400	843	593	701	640
No./Mb	11	23	15	23	19
Length (bp)	6660	14655	10299	12267	11295
Bp/Mb	187	405	267	406	329
Tetra					
No.	19	38	24	22	42
No./Mb	1	1	1	1	1
Length (bp)	392	812	568	460	940
Bp/Mb	11	22	15	15	27
Penta					
No.	14	10	15	10	21
No./Mb	0	0	0	0	1
Length (bp)	425	300	450	270	565
Bp/Mb	12	8	12	9	16
Hexa					
No.	30	23	28	17	88
No./Mb	1	1	1	1	3
Length (bp)	966	1530	3132	648	5430
Bp/Mb	27	42	81	21	158

**Table 5:** The longest microsatellite motifs in the five edible fungal genomes.

	Longest microsatellites		
	Motif	Repeats	Size
*V. volvacea*	A/T	61	61
*C. cinereus *	AACCCT/ATTGGG	34	204
*S. commune *	AACCCT/ATTGGG	36	216
*A. bisporus *	AG/CT	34	68
*P. ostreatus *	AACCCT/ATTGGG	38	228
