# Supplementary material for: Microsatellites in the Genome of the Edible Mushroom, Volvariella volvacea
Source: Biomed Res Int. 2014 Jan 19;2014:281912. doi: 10.1155/2014/281912 (PMC3915763; doi:10.1155/2014/281912)
Supplement: Supplementary file 1 — The detailed information for the fungal genomes involved in the current study, the primers randomly selected from V. volvacea genome, and significant microsatellites in V. volvacea were available in the Supplementary Materials. [file 281912.f1.pdf]

Table S1

List of analyzed edible fungal genomes their web sites.

Organism	Public web site
Volvariella volvacea V23	http://genome.jgi-psf.org/pages/dynamicOrganismDownload.jsf?organism=Volvo1
Coprinus cinereus	http://www.broadinstitute.org/annotation/genome/coprinus_cinereus/MultiDownloads.html
Schizophyllum commune H4-8 v2.0	http://genome.jgi-psf.org/Schco2/Schco2.download.html
Agaricus bisporus var bisporus (H97) v2.0	http://genome.jgi-psf.org/Agabi_varbisH97_2/Agabi_varbisH97_2.download.html
Pleurotus ostreatus PC15 v2.0	http://genome.jgi-psf.org/PleosPC15_2/PleosPC15_2.download.html

Table S2

Set of primers corresponding to the 100 microsatellite locus randomly selected from *V. volvacea* genome.

Microsatellite	Primer sequence	Position on *V. volvacea* genome
(TGCTGA)5	(F)ACGACGCGTATTTGTTTTCC	Scaffold1_431928-431957
	(R)GCAGTTTGACCCCACTCATT	
(ACGAAG)6	(F)GAAAAGCGTGTGGTTGGATT	Scaffold1_612925-612960
	(R)TACTCGTCCTCGTCCTCGTT	
(TCC)8	(F)ACTCCTCTGCAGCTTTCTCG	Scaffold1_1065146-1065169
	(R)GGTTTACGAGCTCTTGGCTG	
(GTG)6	(F)AATGAAGCAACAGATTGGGG	Scaffold1_1906218-1906235
	(R)GGGTGTGACAGAACTGCCTT	
(GTT)6	(F)GTCGAGGTTGATGTCGAGGT	Scaffold1_2488335-2488352
	(R)GGCCTAATTGTCTCGAGTGC	
(CTG)7	(F)ATCCTTGTCGGAAGTTGGTG	Scaffold1_2687794-2687814
	(R)GGAAAAGGAACGGAAGAAGG	
(TGG)10	(F)TGATGGTGTAGCAGTGGCAT	Scaffold1_3470394-3470423
	(R)CAGTCACAGTCTGGTGGTGC	
(TGCC)6	(F)GGTCAGAGGCTTGTACTCGG	Scaffold1_3819500-3819523
	(R)TCTCTTTGCTCGAGCTCCTC	
(TCCGAG)5	(F)CTTACTCTGCTTCGCTGCCT	Scaffold2_348331-348360
	(R)CGAAGACGAAGAGGATGAGG	
(T)15	(F)TTCAGAAATCGAATGGAGGG	Scaffold2_629590-629604
	(R)TCCATCTCGTACGAAGACCC	
(A)38	(F)GTCGCTGATTGTCCCAGAAT	Scaffold2_1281474-1281511
	(R)TATTGCGTAGTGTGCCCAAA	
(CGTGTG)5	(F)AACACTCGACCTGACCCAAC	Scaffold2_1299385-1299414
	(R)ATGCAAGGTCAAGGGACAAC	
(TTC)6	(F)GTTTGCCTGCTTCACCATCT	Scaffold2_1901177-1901194
	(R)CAATGGTACCCTGCTCGAAT	
(GTAGT)6	(F)GCCCCACCATGGTAGTGTAG	Scaffold2_2144371-2144400
	(R)GGGAGAGGGAGGAAGAGAGA	
(AAAATA)5	(F)GATTTCCAAAGGTGGCTCAA	Scaffold2_2549073-2549102
	(R)CTTTCTGCATGAGTTGTGGC	
(CCT)6	(F)CTATCCCCACCAGCATCACT	Scaffold3_131152-131169
	(R)GTGCACCAGACCCTGGTAAT	
(G)16	(F)CGTCACCTGCGTTCACTCTA	Scaffold3_631540-631555
	(R)AATTTCAAACACCCCAACCA	
(GAG)13	(F)AGTGCTGGACGACATGACTG	Scaffold3_1135265-1135303
	(R)AGGTGGTAGAGGCATGGTTG	
(AAGGAC)9	(F)ACCAATGGGTCTAGGTGCTG	Scaffold3_1846823-1846876
	(R)TGCCTCTCATCAAGATCACG	

(GTC)7	(F)AAGCTGTACGAGAAGGCGAC	Scaffold3_2393769-2393789
	(R)AGCGACATAAGCCAACATCC	
(CCT)7	(F)CCGAGCTCGCATACTCTTCT	Scaffold4_381002-381022
	(R)TAGAGGATTGTACGGGGCAG	
(CGAAA)6	(F)AAGCCCTACTCCAGCTCCTC	Scaffold4_570255-570284
	(R)CAACTATTTTCCGCCACCAT	
(ACCG)5	(F)GATCAGGCCCACACAAAAGT	Scaffold4_1042332-1042351
	(R)ATCGAATAACTGGACCGACG	
(A)23	(F)AATTGGGACATTTGAGGCAC	Scaffold4_1688293-1688315
	(R)CCTCGCTTCTCAAGGCAATA	
(CACGA)5	(F)GCTGATCACCGTTTTCGTTT	Scaffold4_2262668-2262692
	(R)GTTGATGGGCACGGTAGACT	
(T)16	(F)TTGAAGCAAATGTTTGCGAG	Scaffold5_268766-268781
	(R)CTTGCATGATCCCATCCTTT	
(GCC)6	(F)GATCCATTAATGATGCGGCT	Scaffold5_982489-982506
	(R)CTTGCTTTGGCTCAAGGTTC	
(AT)10	(F)GATGAAGGTGTCGGTGGAAT	Scaffold5_1073542-1073561
	(R)TTGCGTTTTCGAACTGTCTG	
(GAC)7	(F)TCTACGCAGGAACACACTGG	Scaffold5_1598143-1598163
	(R)AAAATTGCTGCAAAGTGGCT	
(A)26	(F)TATACCATTGCTGGGGCATT	Scaffold6_315238-315263
	(R)AGTGGGACTCTGCAAGGCTA	
(TGGT)6	(F)GGAAGGAAGGGGTGAGAGTC	Scaffold6_765056-765079
	(R)ACGCTGGACCCCTAAAGAAT	
(CTTCGA)5	(F)GCGTGAATGCCGGTACTAAT	Scaffold6_1336082-1336111
	(R)CCGGTAGTTGGACTCCAGAA	
(CAG)8	(F)TCACCATCAGTTCCAGATGC	Scaffold7_119885-119908
	(R)TCACGATTTCACGCTCTCAC	
(GAC)8	(F)ATATCCTTCGCGATGACCTG	Scaffold7_320416-320439
	(R)GTACTCCAGCACGGCATCTT	
(T)61	(F)CTTTCTGGGCTTTCGAGGTT	Scaffold7_962608-962668
	(R)GGCAAAGAAACGAAATGGAA	
(GTG)9	(F)TGCGATAGCACTCAAAGGTG	Scaffold8_127150-127176
	(R)GGCGTTCTATTCGTTCCAAA	
(TACGAA)5	(F)GAGCCGTCACATCTTGACCT	Scaffold8_532402-532431
	(R)CCATTCCTGCTTCTTCTTCG	
(GTC)7	(F)ATACGGGATACCGCTCTGTG	Scaffold8_1157968-1157988
	(R)ACCTCCGTTTTGGTTCACTG	
(TTTCTT)5	(F)TGGCACTTGTCACTCCTCTG	Scaffold9_434842-434871
	(R)AGACCTCTGAAGGTGGCTCA	
(GGATAT)5	(F)AGAGACCGATGGCAGAAAGA	Scaffold9_814118-814147
	(R)TCCTTCCATACACTCCCTGC	
(TCTTCG)5	(F)TTTCGGACAGGAGAGAGTCG	Scaffold10_162541-162570

	(R)TGGAGAAGAGGCAGGGTATG	
(GCACAA)5	(F)CGACTGCTCAAGTACACCCA	Scaffold10_419127-419156
	(R)CTCCTCCTGTGCTTCGACTC	
(TGCTGG)5	(F)TATACGGCAGATTTGTTGCG	Scaffold11_139461-139490
	(R)AGCAGCAGCAGGGAATGTAT	
(CCGCAC)6	(F)CGTCTACATCCACAACACCG	Scaffold11_433434-433469
	(R)CTGTCGTCGTCTTCGTCTTG	
(GGT)5	(F)ATCGTCTACGGAGATCGGTG	Scaffold11_608487-608501
	(R)CTACCACCACCAAGTCCACA	
(GTGCTT)5	(F)CCTTGTTCCGTTTGCTTGTT	Scaffold12_249930-249959
	(R)AAACTAGTCCCAAGGCGGAT	
(GAGTGC)5	(F)GAGCGATACTGCTGCTGTTG	Scaffold12_784704-784733
	(R)CCAACTCAAACAGCTCCCAT	
(CCGG)5	(F)CTTGCCTTGGTCAAATCCAC	Scaffold13_157743-157762
	(R)GCAGGCTAAAGGGGTTCTTC	
(TTTTC)5	(F)TCTTGAGTGGCTATTGTCAGGA	Scaffold13_687861-687885
	(R)AATGACGCCACCGAGAATAC	
(GTG)7	(F)TTTATGACTGGCTGTGGCTG	Scaffold14_45258-45278
	(R)CGCAGGAGAGATGCATACAA	
(GT)8	(F)CAACCTCCTCTTCCTCCCTC	Scaffold14_562610-562625
	(R)TCCTGCCCACACAGTCATAA	
(CAC)14	(F)TTTGCATCTAATGACGCCAC	Scaffold15_112434-112475
	(R)TTGTCTTTGAACATTCACATTGC	
(TTTTC)5	(F)CCTGGGCTCTGTCACTGTCT	Scaffold15_631004-631028
	(R)TAAGCAAGTCACGTGGTCCC	
(GTTCTG)5	(F)GGCTGATGACTTGGGTGACT	Scaffold16_224142-224171
	(R)AGATGATGATGAGGATCGGC	
(AAG)8	(F)AGGAGGAGGAAGAGGCAAAG	Scaffold16_721355-721378
	(R)ACAACGAGCGTCATAAGCCT	
(TTTCTT)5	(F)AAATACAGGCCTGGCAAGTG	Scaffold17_318630-318659
	(R)CACATGGTCCTTGGTGCTTA	
(T)20	(F)CCACCACCTCACACTATCCC	Scaffold17_615280-615299
	(R)GGGTGAGCAATCGGAAGATA	
(A)22	(F)GTCTCCCGATGTGTTCTGGT	Scaffold18_145216-145237
	(R)CACGCGTCTGCAGGTATAGA	
(ATT)8	(F)ACCGCCCCTTTCTTAATGTT	Scaffold18_541805-541828
	(R)ATCTGCAACAACGAACCACA	
(T)33	(F)TCTTAGGGTCGACTCGGATG	Scaffold19_440824-440856
	(R)AGCACATGGGAACCAGAAAG	
(ACC)9	(F)CGTCTTCACCTCTACCCCAA	Scaffold19_646423-646449
	(R)GCTGTGATGGCAGACACACT	
(A)38	(F)CCAAGCAAGGAACAAGGAGA	Scaffold20_2127-2164
	(R)GGCATTTCTGCCAAAGAGTC	

(TCG)7	(F)CTGTCCACCACCTCAATCG	Scaffold20_605184-605204
	(R)ACGTCTCATTTCTTCACGGG	
(T)26	(F)AAGGAAACATGCCCACAAAA	Scaffold21_20097-20122
	(R)TGCCTATGTATTCTTGCGACC	
(TCG)6	(F)CAGGAACAGCCCATCTTCTC	Scaffold21_169819-169836
	(R)GTATGTGCATCTCGCTTGGA	
(GAC)6	(F)ACGGAGCAGGTCGAGAGATA	Scaffold22_303125-303142
	(R)ATCAAACCAAAGAACAGCGG	
(TGC)7	(F)AGTTGTTGTCGTTGCTGACG	Scaffold22_601066-601086
	(R)AAACCAGAACCTGCAACACC	
(GCA)6	(F)ATGTGGCTAACTCCTGGGTG	Scaffold23_499139-499156
	(R)TGACGAGTAGCTGGTGATGC	
(T)21	(F)ATGTTGTCGTGTCGTGTCGT	Scaffold23_556485-556505
	(R)TTGATACTGCCTACCCCAGC	
(ACG)8	(F)GTGGGGATAGAACTGGGGTT	Scaffold24_291636-291659
	(R)GGCTTCAATCCCTAGATGGC	
(A)19	(F)TGCTCTTACGGTCTCGGTCT	Scaffold24_413622-413640
	(R)GGTTTGCGATTATGGTGGTC	
(T)22	(F)GCAGGAAGTCCTCCAAAGTG	Scaffold25_80676-80697
	(R)GGGTTCGGGTATTCCTTGAT	
(GTCTT)5	(F)CACGTTTTTCGTGTTTCGTG	Scaffold25_335713-335737
	(R)AAACAGTCGAGACGGAGACG	
(TCA)8	(F)GCACTGCTTTCCTCATCTCC	Scaffold26_284037-284060
	(R)GCCTATGATATGGAGCGAGC	
(GTG)12	(F)AGTGCGACGAGCCCTAGTTA	Scaffold27_391371-391406
	(R)AGATTCTCCCTGACCCCATC	
(ACCAAA)5	(F)TCAGCGTCCATTCACTCAAG	Scaffold28_255725-255754
	(R)GCTCTGCAGGAGGCAATAAC	
(A)21	(F)AGGCGAGGAAAAATCACCTT	Scaffold29_291594-291614
	(R)GAACAGTTCTGCTGGGGTGT	
(AAAATA)7	(F)GATTTCCAAAGGTGGCTCAA	Scaffold30_2671-2712
	(R)ATGCACTGCTCAAGATGCAC	
(ATCTAC)5	(F)ATGTACAGGATTTGCCGCTC	Scaffold31_85372-85401
	(R)CCCGCTGAAATTAACGAAAA	
(TGACC)7	(F)CTTTCAGGAAGAGCCCAGC	Scaffold32_217019-217053
	(R)TGAAGGGCTTGAAGGAGAAA	
(CCTTT)5	(F)GTGGCTGAGTGATTTCCCAT	Scaffold33_62403-62427
	(R)TTCAACAGTGCCCAGTTACG	
(GTG)7	(F)ATAACCGGAAGCAGAAGCAG	Scaffold34_109975-109995
	(R)TTCTATCACAACAAGCCCCC	
(CCGAGT)5	(F)ATATCTGGCTTCGACGGTTG	Scaffold35_63354-63383
	(R)GATGTCCAAGTCCGAGTGGT	
(TTTCTC)5	(F)GCTGGCTGCATAAACGGTAT	Scaffold36_143288-143317

	(R)AGGGAAATCGAAAGCGGTAT	
(A)35	(F)TCCAAGCGTCTATCAATCCC	Scaffold37_171896-171930
	(R)CTTTTCGGCGACTTTTTCTG	
(AAT)8	(F)TCAAGAGACAAAAAGTGAGCAAA	Scaffold38_108483-108506
	(R)TGTTGACTGCCCACATTGAT	
(GGATTG)5	(F)GCGTCTTGGAGAAGCAAAAC	Scaffold39_179253-179282
	(R)AATCGTGCACAAAGGAAACC	
(G)16	(F)TTATGACCCCTTCCAGATCG	Scaffold40_34075-34090
	(R)GGTTGGAAGCAGACGAAGAG	
(CGT)6	(F)AAAAGCGCCCATTTGAACTT	Scaffold41_115044-115061
	(R)CGTCAACTACCTCCCCAACA	
(CAC)6	(F)GTCATCTGACCGCCTGGTAT	Scaffold42_44443-44460
	(R)TTGGGTTTAAGCATTCTCGG	
(AT)8	(F)TGCTAGCCATGGCCTACTCT	Scaffold43_117069-117084
	(R)AGTCAAGTTTAGGGCTGCCA	
(TGA)7	(F)TTCTTTTTGATTATGGCGGC	Scaffold44_96228-96248
	(R)AACTCCACAACCACTACGCC	
(ACC)6	(F)GTTGGCACCACTACATCACG	Scaffold45_109122-109139
	(R)GGTGGGCTATCAGCATCATT	
(T)12	(F)CCAAGGAACTTCACACCAAA	Scaffold46_3720-3731
	(R)GTGACAGGGCAACAATTCAG	
(CCCATA)5	(F)GTGTTCGTACTGGGCTTGGT	Scaffold47_20934-20963
	(R)TCAGAATGGTATGCGGATCA	
(T)10	(F)TTGGTGCATCTCCATCAAAA	Scaffold48_59215-59224
	(R)CAAACGTCAAATGAACGTGG	
(TC)8	(F)GGTTCTCTCTCCCTGCCTCT	Scaffold49_3987-4002
	(R)TCAAGCTGACAAGGAACACG	
(A)12	(F)ATTTTTAAACGGAGGGGTGG	Scaffold51_25191-25202
	(R)TAGCCCATGTGGACAAGACA	
(A)20	(F)TTTTAGGCTGCTCATGGCTT	Scaffold56_3669-3688
	(R)AGCTTGAGGTTGATGGGATG	
(TCG)5	(F)GTGATTGCTACGGGCTTGAT	Scaffold59_4798-4812
	(R)TCGATGGTGAAATGCGAATA	

Table S3

Microsatellites in the gene models with some specialized function in *V. volvacea*.

Gene Product	Number of gene	Location
CYP547B1	1	Intron
CYP5080B3b	1	Intron
CYP627A1	1	Intron
Ectomycorrhiza-upregulated GH16 glucan endo-1,3-beta-glucosidase precursor	1	Intron
GH16 beta-1,3-glucan recognition protein	1	CDS, Intron×2
CAMK/CAMK1 protein kinase	1	CDS×4
CAMK/CAMKL/Kin1 protein kinase	1	CDS
CAMK/CAMKL/Kin4 protein kinase	1	CDS
CMGC/SRPK protein kinase	1	CDS
Hypothetical kinase	1	CDS
Mps one binder kinase activator-like 4	1	CDS
CAMKK/ELM protein kinase	1	CDS
HAL protein kinase	1	5'UTR
RAN protein kinase	1	Intron
Phosphatidylinositol phosphate kinase PIPK5	1	3'UTR
STE/STE11/cdc15 protein kinase	1	CDS
Candidate aryl-alcohol dehydrogenase	1	Intron
Dehydrogenase E1 and transketolase domain-containing protein 1	1	Intron, Intron
Dihydrolipoamide dehydrogenase	1	Intron
IMP dehydrogenase	1	CDS, Intron
Pyruvate dehydrogenase	1	CDS
Mitochondrial half-size ABC transporter	1	CDS
Multidrug resistance-associated ABC transporter	1	Intron
OPT oligopeptide transporter	1	Intron
Pleiotropic drug resistance ABC transporter	1	Intron
Sodium transport ATPase	1	Intron
Urea transporter	1	3'UTR
Vacuolar amino acid transporter 4	1	Intron×2
ZIP-like iron-zinc transporter	1	CDS
60S ribosomal protein L18-B	1	3'UTR
ABC domain-containing protein	1	CDS
Adaptor protein	1	CDS
Adenylate cyclase	1	CDS
ADP-ribosylation factor	1	CDS
Aminopeptidase	1	Intron
AMP dependent CoA ligase	1	Intron

Ankyrin repeat protein	1	CDS
Aspartic peptidase A1	1	3'UTR
ATP-dependent DNA helicase MER3	1	Intron
ATP citrate lyase isoform 2	1	3'UTR
Beta-tubulin 2 tubb2	1	Intron
Beta-xylosidase	1	CDS
C-4 methyl sterol oxidase	1	Intron
Calnexin	1	3'UTR
Carnitine/acyl carnitine carrier	1	Intron
CDC5	1	Intron
Clathrin-coated vesicle protein	1	Intron
Coiled-coil protein	1	CDS
COP8	1	CDS
Copper-fist transcription factor	1	5'UTR
Cytoplasmic protein	2	Intron×2
Deoxyuridine 5'-triphosphate nucleotidohydrolase Dut	1	CDS
Dienelactone hydrolase endo-1,3,1,4-beta-D-glucanase	1	Intron
DNA helicase	1	CDS
DNA repair and recombination protein pif1	1	Intron
Endoplasmic reticulum protein	1	3'UTR
Epsin domain-containing protein	1	CDS×2
Exo-beta-1,3-glucanase	1	Intron
Exosome component Rrp46	1	CDS
Expansin family protein	1	CDS×2
Expressed protein	2	CDS×2
Extracellular triacylglycerol lipase precursor	1	Intron
Fructose-bisphosphate aldolase	1	CDS, Intron
Glutamate-rich WD repeat containing	1	CDS
Glycoside hydrolase family 10 and carbohydrate-binding module family 1 protein	1	Intron
Glycoside hydrolase family 13 protein	1	CDS
Glycoside hydrolase family 15 protein	1	Intron
Glycoside hydrolase family 16 protein	1	Intron×2
Glycoside hydrolase family 18 protein	1	CDS, Intron
Glycoside hydrolase family 35 protein	1	Intron
Glycoside hydrolase family 38 protein	1	Intron
Glycoside hydrolase family 92 protein	1	Intron
Glycosyltransferase family 2 protein	2	CDS
Glyoxal oxidase	2	3'UTR
H3	1	Intron
Heparinase II/III family protein	1	Intron

Heterotrimeric G protein alpha subunit B	1	Intron×2
High-glucose-regulated protein 8	1	Intron
Histone acetyltransferase mst2	1	CDS
Histone deacetylase complex, catalytic component RPD3	1	Intron
HNWD1	1	Intron
Iron sulfur assembly protein 1	1	Intron
L-ascorbate oxidase	1	3'UTR
Laccase 1	1	3'UTR
Laccase 3	1	3'UTR
Laccase 6	1	Intron
Lipase	1	CDS×2
Lipase 2	1	CDS×2, Intron
Long-chain-fatty-acid-CoA ligase	1	3'UTR
Metalloprotease	2	Intron×2
Metalloprotease ATP23	1	3'UTR
MIP protein	1	CDS
Mitochondrial chaperone bcs1	1	Intron
Mitochondrial genome maintenance protein	1	CDS
Modular protein with glycoside hydrolase family 13 and glycosyltransferase family 5 domains	1	Intron
NADH-quinone oxidoreductase	1	Intron
Nonribosomal peptide synthetase 12	1	Intron
NUC153 and WD40 repeat-containing nucleolar rRNA processing-related protein	1	Intron
Nuclear condensin complex protein	1	Intron
Nuclear protein	1	3'UTR
Nucleolar protein	1	CDS
NWD2	1	Intron
Peptidase 1	1	Intron
Phosphatidylserine synthase 2	1	Intron
Plant senescence-associated protein	1	CDS, Intron
Pol-like protein Pol-2	1	CDS
Polyprotein	1	Intron
Polysaccharide synthase Cps1p	1	3'UTR
Potassium/sodium efflux P-type ATPase	1	CDS×3, Intron
Pre-mRNA-processing ATP-dependent RNA helicase PRP5	1	Intron
Protoporphyrinogen oxidase	1	Intron
RAB-protein geranylgeranyltransferase	1	3'UTR
Ras protein	1	Intron
Receptor expression-enhancing protein 4	1	CDS

Related actin-binding protein	1	CDS
Retrotransposable element Tf2 155 kDa protein type 1-like	1	Intron
Rho GTPase activating protein 22	1	CDS
ROT1 protein	1	3'UTR
RRM/R3H domain-containing protein	1	Intron
Sec7 guanine nucleotide exchange factor	1	3'UTR
Serine palmitoyltransferase 2	1	CDS
Serine protease inhibitor	1	Intron
SH3 domain-containing protein	1	3'UTR, Intron
Siderophore biosynthesis regulatory protein	1	Intron
Sim15	1	Intron
Sodium transport ATPase	1	Intron
Splicing factor SF1	1	Intron
STE-12 alpha	1	Intron
STE20	1	CDS
Structural maintenance of chromosome protein 3	1	Intron
Tetraspanin Tsp2	2	CDS
Trehalase	1	Intron
Ubiquitin-conjugating enzyme E2	1	CDS
Ubiquitin C-terminal hydrolase	1	CDS
Unnamed protein product	1	5'UTR
Vacuolar DHA amino acid exporter	1	Intron
Vacuolar membrane protein	1	CDS
Vacuole protein	1	Intron
XPG I-region protein	1	CDS
Xylanase	1	CDS
ZYRO0D15356p	1	CDS
